# Supplementary figures and images for: Dissection of Metabolome and Transcriptome—Insights into Capsaicin and Flavonoid Accumulation in Two Typical Yunnan Xiaomila Fruits
Source: Int J Mol Sci. 2024 Jul 16;25(14):7761. doi: 10.3390/ijms25147761 (PMC11276673; doi:10.3390/ijms25147761)

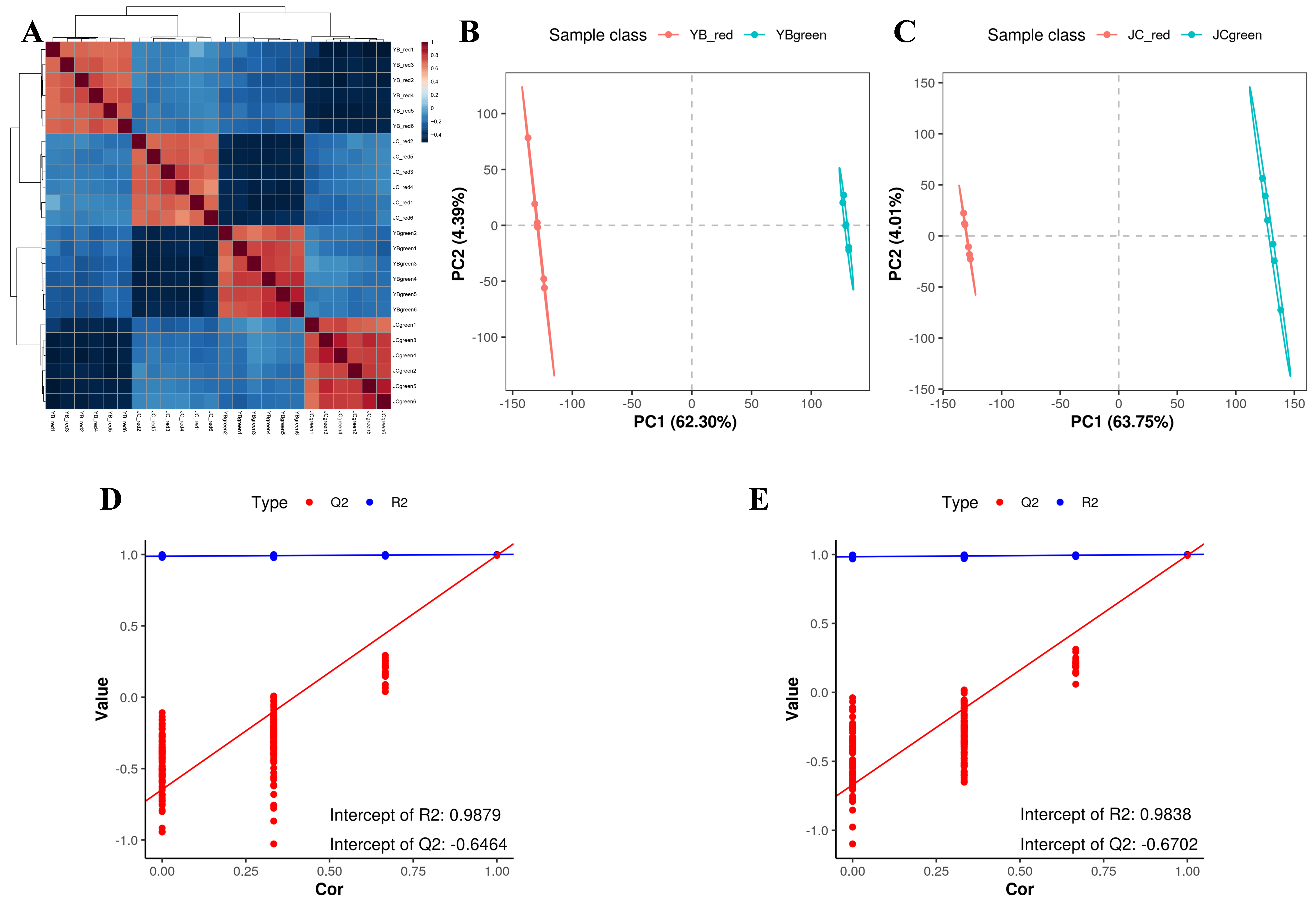

Supplement: Supplementary file 1 [file ijms-25-07761-s001.zip › Figure S1. The global analysis of metabolic composition in green and red mature stages of both pepper cultivars.tif]

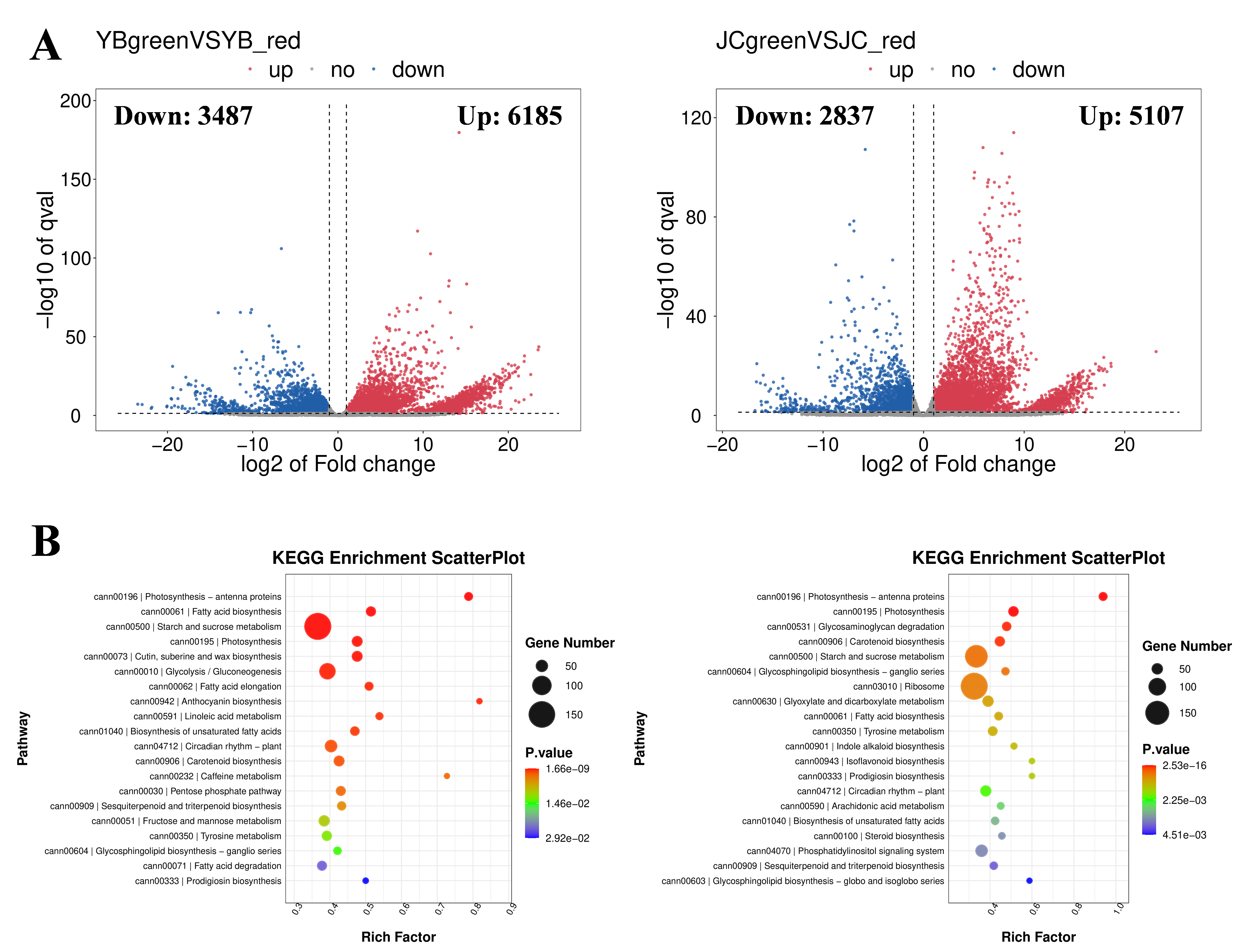

Supplement: Supplementary file 1 [file ijms-25-07761-s001.zip › Figure S2. The global analysis of transcriptome composition in pairwise comparison of YB-green vs. YB-red and JC-green vs. JC-red.tif]
